# Flexible highly-sensitive pressure sensor based on rGO/Fe nanowires composites for wearable human health detection

**DOI:** 10.3389/fchem.2024.1477651

**Published:** 2024-10-30

**Authors:** Liang Cao, Rui Wu, Hong Xiang, Xiaodong Wu, Xiaoyan Hu, Gaohui He, Yingang Gui

**Affiliations:** ^1^ College of Engineering and Technology, Southwest University, Chongqing, China; ^2^ Chongqing Urban Power Supply Branch, State Grid Chongqing Electric Power Company, Chongqing, China; ^3^ State Grid Electric Power Research Institute of Chongqing Electric Power Company, Chongqing, China

**Keywords:** flexible pressure sensors, rGO/Fe NWs composites, microstructural design, human health detection, wearable detection

## Abstract

Flexible pressure sensors applied in wearable detection often face challenges, such as low sensitivity, large device size, poor flexibility, and long response time. This study aims to design and develop high-performance pressure-sensitive materials for wearable human detection applications. Using a sensitive layer composite and microstructural design, rGO/Fe nanowires (NWs) composites were proposed as the pressure-sensitive material. This approach yields a compact sensor with high flexibility, good mechanical properties, and excellent sensing performance. Firstly, rGO/Fe NWs composites were prepared by the Hummers method and an *in situ* reduction technique under a magnetic field. Secondly, the structural design, component construction, and sensing mechanism of the sensors were thoroughly investigated. Finally, the performance of the flexible pressure sensor was tested, and its application in the wearable field was explored. The results demonstrate that the sensor exhibits excellent performance with a good response to both large and small pressures within the range of 0–30 kPa, providing an effective method for wearable human health detection.

## 1 Introduction

Wearable devices have received extensive attention in recent decades, showcasing great potential for the continuous monitoring of health conditions and body movements (Zheng et al., 2023). Flexible pressure sensor devices with high flexibility, sensitivity, and durability can be used as wearable detection devices for observing pulse, respiration, and limb movement (Sengupta et al., 2019; Zhiwen et al., 2023). However, traditional pressure sensors are typically rigid, making them unsuitable for wearable applications. Therefore, there is an urgent need to develop pressure-sensitive materials with good responsiveness, flexibility, ductility, and biocompatibility (Zhou et al., 2019; Yin et al., 2023; Wang C. et al., 2020).

While wearable human health detection systems based on pressure-sensitive materials have been prepared (Kausar, 2020), current pressure sensors often suffer from large overall size, low flexibility, stiffness, and weak sensing performance, which limit their effectiveness in wearable applications (Xiao et al., 2023; Pang et al., 2018; Chen et al., 2020). Flexible pressure sensors constructed from modified sensitive composite materials, however, offer high sensitivity and short response or recovery times, indicating substantial potential for use in wearable pressure detection (Li G. et al., 2020). Therefore, it is crucial to develop highly sensitive flexible pressure sensors for detecting health conditions and body movements (Gui et al., 2022).

Exploring high-performance pressure-sensitive materials is essential for fabricating effective pressure sensors. Reduced graphene oxide (rGO) not only possesses excellent flexibility and mechanical properties, but also shows strong hydrophilicity due to its functional groups, facilitating the formation of composites with other pressure-sensitive materials to enhance sensor performance (Li W. et al., 2020; Biutty et al., 2020; Chang, 2023). In addition, iron nanowires (Fe NWs) are exemplary pressure-sensitive materials with out-standing pressure-sensitive properties. It has advantages such as low cost, easy preparation, and easy regulation by magnetic fields to meet variable sensing demands (Zhao et al., 2023; Sriphan et al., 2021; Ismail et al., 2023).

The construction of excellent surface-modified microstructures is also crucial for achieving highly sensitive flexible pressure sensors (Bai et al., 2023; Wang et al., 2019). Among the common methods for creating modified microstructures, the silicon wafer template method used by Lee et al. has shown some success but also poses drawbacks such as environment harm from the etching process, the high preparation cost, and complex production process (Lee et al., 2020). For the porous elastomer method used by Ding et al., the raw material for the porous structure preparation was difficult to remove in the subsequent process, which could compromise the sensing performance of the entire flexible pressure sensor device (Ding et al., 2019). Wang et al. used sandpaper template to form a PDMS film with microstructures on both sides (Wang X. L. et al., 2020), paving a promising approach for constructing wearable sensors.

In this study, we developed a flexible pressure sensor based on rGO/Fe NWs composites, featuring compact size, high flexibility, good mechanical properties, and excellent sensing performance through the design of sensitive layer composites and microstructures. The real-time pressure sensing response under different pressure conditions were tested. The detection results indicate that the sensor has good pressure response and flexible properties, broadening its applications in flexible pressure sensors in wearable fields, health management, human activity, and human-computer interaction.

## 2 Materials and experimental methods

### 2.1 Materials

Concentrated sulfuric acid (H_2_SO_4_, ≥98%), concentrated phosphoric acid (H_3_PO_4_, ≥85%), graphite powder (325 mesh), hydrazine hydrate (N_2_H_4_·H_2_O), sodium borohydride (NaBH_4_, ≥98%), anhydrous ethanol (EtOH), n-pentane (C_5_H_12_), ethyl acetate (C_4_H_8_O_2_), polydimethylsiloxane, namely, (C_2_H_6_OSi)_n_, trimethylchlorosilane (C_3_H_9_ClSi), and sodium polystyrene sulfonate (C_8_H_7_NaO_3_S, PSS) were purchased from Chongqing Yuexiang Chemical Co. Potassium permanganate (KMnO_4_), acetone (C_3_H_6_O), toluene (C_7_H_8_) and poly diallyl dimethyl ammonium chloride ((C_8_H_16_NCl)_n_, PDDA) were purchased from Chengdu Kolon Chemical Co. Hydrogen peroxide (H_2_O_2_, ≥30%) and ferrous sulfate heptahydrate (FeSO_4_·7H_2_O, ≥99%) were purchased from Chongqing Xingguang Chemical Glass Company. Hydrochloric acid (HCl) was purchased from Chongqing Taixin Chemical Co. The silane coupling agent (C_9_H_23_NO_3_Si, APTES) was purchased from Chongqing Kangni Trading Co. Ultra-pure water with a resistivity of >18.2 MΩ cm was used for the experiments.

### 2.2 Preparation of rGO

The GO (graphene oxide) nanomaterial was prepared by the Hummers method. First, 6.67 mL H_2_SO_4_ and 60 mL H_3_PO_4_ were mixed at 5°C. Then the acid mixture was added into a beaker containing 0.5 g graphite powder and 3 g KMnO_4_, and stirred at 50°C for 6 h/7 h/8 h/9 h H_2_O_2_ was added until the solution turned bright yellow and no bubbles were formed (Jiang et al., 2022). The reaction system was heated and stirred for 3 h and then diluted with ultra-pure water. The resulting reaction product was centrifuged five times at 8,000 rpm for 5 min each time. The total amount of 20 mL was divided into four equal portions. GO and N_2_H_4_·H_2_O were added at the mass ratios of 10:1, 10:5, 10:7, and 10:10 (Li et al., 2021), respectively to reduce the Go at 90°C for 1 h, then filtered and placed in the oven at 30°C for 36 h to obtain rGO.

### 2.3 Preparation of Fe NWs

An appropriate amount of FeSO_4_·7H_2_O was dissolved in ultra-pure water and stirred thoroughly to obtain a 0.1 mol/L solution with a clear bright blue color. In a low-temperature water bath, an appropriate amount of NaBH_4_ was made into a solution in ultra-pure water and stirred to obtain a solution of 1.4 mol/L. NaBH_4_ is susceptible to deliquescence, so it is important to use it as soon as possible after opening. The FeSO_4_·7H_2_O solution was placed into a PVC reaction vessel with a pair of ferrite magnets on either side to create a magnetic field of 100 mT in the transverse direction of the vessel (Yang et al., 2022). The NaBH_4_ solution was drawn and sprayed vertically into the vessel at a rate of 20 mL/min with a syringe at room temperature. Then the black loose deposit was removed with a NdFeB magnet. To eliminate impurities formed during the reaction, the sediment was centrifuged three times with ultra-pure water and EtOH respectively to obtain Fe NWs.

### 2.4 Construction of protective layers with microstructure

First, PDMS was prepared by mixed (C_2_H_6_OSi)_n_ and APTES at the mass ratio of 10:1. Second, a PDMS/C_4_H_8_O_2_ mixture (V_PDMS_: V_C4H8O2_ = 7:3) was sealed in a beaker by a cling film, and stirred magnetically for 30 min (Li et al., 2019). Third, the PDMS/C_4_H_8_O_2_ mixture was spread on a glass plate rinsed several times with C_7_H_8_ and EtOH and scraped with a Meyer bar. Sandpaper of 100, 180, 240, and 500 grit was selected and 2 cm × 2 cm was cut as a template. The sandpaper was soaked in C_3_H_9_ClSi/C_7_H_8_ (V_C3H9ClSi_: V_C7H8_ = 1:4) release agent for 30 min to obtain a sandpaper template (Zhang, 2023). PDMS was applied to the surface of the sandpaper and cured at 80°C for 5 h. The PDMS film was removed from the fluted structure with tweezers and also stripped. The PDMS/C_4_H_8_O_2_ hybrid system was applied on top and cured at 80°C for 5 h to obtain the modified microstructure layer.

### 2.5 Construction of a composite sensitive layer

The cotton and polyester fabric carriers were ultrasonically cleaned with EtOH and ultra-pure water, respectively, and then impregnated in beakers containing PDDA (1.5 wt%) and PSS (0.3 wt%) for 10 min (Zhong et al., 2016). The roughly treated carriers were surface-modified by heating at 100°C for 10 min in a hydrothermal reactor with APTES (Zhong et al., 2023). Contact ultrasonic dispersion was performed on the rGO solution and non-contact ultrasonic dispersion was performed on the Fe NWs solution. 5 mL C_5_H_12_ was added to 5 mL rGO solution at a concentration of 0.01 mg/mL, 3 mL C_3_H_6_O was injected and immediately heated to 120°C for 1 min, and then cooled at room temperature for 3 min to allow the self-assembly of rGO on the carrier to form. The carrier was soaked in EtOH for 2–3 min, the carrier was clamped on the sand core with tweezers, and the Fe NWs solution was poured for *in situ* preparation of rGO/Fe nanowires composites, the opening and closing of the vacuum extraction device was controlled to allow the uniform deposition of Fe NWs *in situ* attach on the carrier to obtain the composite sensitive layer.

### 2.6 Assembling the flexible pressure sensor

A packaging mold of 2 cm × 2 cm× 0.2 mm was established, as its size is small enough to prepared a miniaturized flexible pressure sensor applying in wearable sensing field. The packaging mold was obtained by engraving an acrylic plate with a laser cutting machine. The mold was polished with sandpaper, and cleaned with ultra-pure water, then dried at 35°C. The transmission electrode layer was composed of an interdigital electrode cut out by copper foil glue and conductive silver paste. The protective layer, modified microstructure layer, composite sensitive layer, transmission electrode layer, modified microstructure layer, and the protective layer were placed into the mold in order. The rGO/Fe NWs composites based flexible pressure sensor was obtained by pouring into the PDMS/C_4_H_8_O_2_ mixture system and drying at 70°C.

### 2.7 Characterization

The structural morphology of GO was characterized by XRD (DX-2700) and the microscopic morphology of GO, rGO, Fe NWs, and rGO/Fe NWs composites sensitive layers was characterized by SEM. The mechanical properties of the flexible pressure sensors were measured by applying external loads using stepper motors (RXPN60) and a push-pull gauge (HP-500). The electrical signals of the flexible pressure sensors were obtained by using an electrostatic meter (Keithley 6514).

## 3 Results and discussion

### 3.1 Microstructure and characterization of pressure-sensitive materials

**FIGURE 1:**
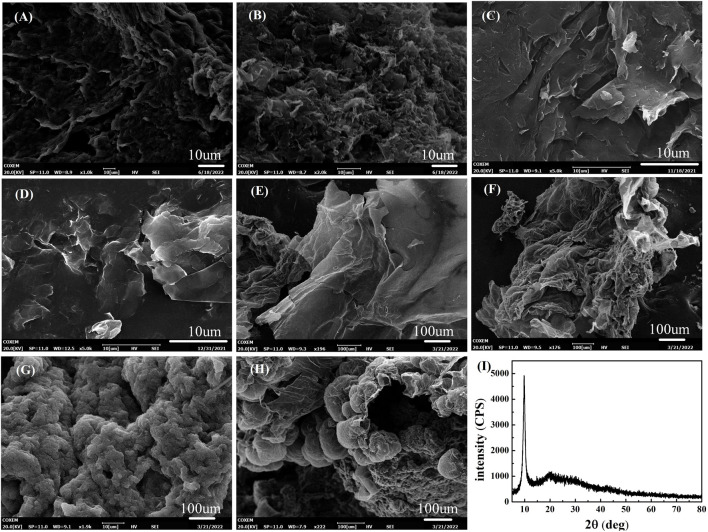
Effect of reaction time on morphologies of GO, including **(A)** 6h, **(B)** 7 h, **(C)** 8 h, and **(D)** 9 h; Effect of reduction ratio on morphologies of rGO, including **(E)** 10:1, **(F)** 10:5, **(G)** 10:7, and **(H)** 10:10; **(I)** XRD pattern of GO.

Figures 1A–D show the SEM images of GO at different reaction times of 6 h, 7 h, 8 h, and 9 h, respectively. In Figures 1A, B, it could be clearly seen that the edges of the GO were curled and wrinkled due to oxidation, and the GO demonstrated a completely different state compared with graphite as the reaction time increased. For reaction at 8 h as shown in Figure 1C, a clear layered structure of the GO reaction appeared, but these layers were still stacked together, indicating that the GO was not completely exfoliated. After 9 h reaction as illustrated by Figure 1D, it was found that the GO exfoliation did not increase with time, indicating that the oxidation time of GO finished. Meanwhile, XRD characterization analysis could be used to accurately characterize the changes in the sample lattice. The XRD graph in Figure 1I shows that the characteristic diffraction peaks emerged around 2*θ* = 10.70° for GO (001) crystal plane, indicating that the graphite powder crystal structure was successfully transformed into that of GO by the oxidation reaction. Figures 1E–H show the rGO morphology prepared by GO and N_2_H_4_·H_2_O with the mass ratio of 10:1, 10:5, 10:7, and 10:10, respectively. It could be clearly observed that a certain degree of stacking occurred between the rGO layer in Figure 1E. The rGO layer in Figure 1F shows an obvious exfoliated morphology of rGO. rGO obtained in Figures 1G, H show a disordered state with over-lapping layers, entanglement, as well as aggregation, and the curved sections reduced the surface energy. In summary, a mass ratio of 10:5 between GO and N_2_H_4_·H_2_O provided the best morphology of rGO.

**FIGURE 2:**
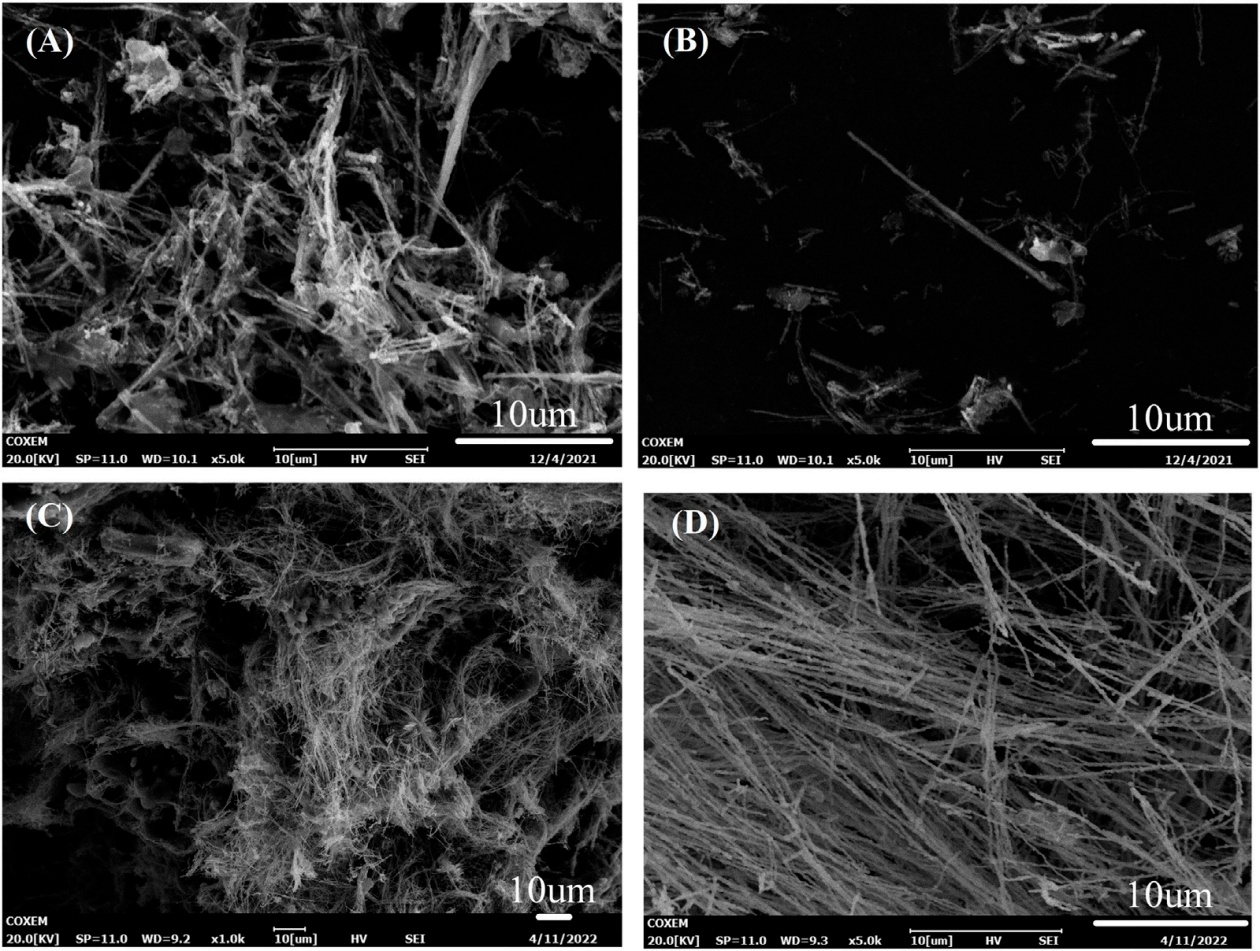
Morphology of Fe NWs at different regions, **(A)** region 1, and **(B)** region 2; SEM images of Fe NWs at different centrifugal speeds, including **(C)** 4,000 rpm, and **(D)** 2000 rpm.

The microstructure of the synthesized Fe NWs was observed by SEM, as shown in Figures 2A, B. It was observed that Fe NWs in Figure 2A were formed by a combination of several iron cores, and their distribution was staggered, which confirmed the growth prediction of Fe NWs. In combination with the morphology of Fe NWs in Figure 2B, it could be found that Fe NWs were not evenly distributed, which included some aggregation of Fe NWs and single-digit Fe nanoparticles. In addition, a large number of impurities were mixed in it, which would affect the purity of Fe NWs and further affect the sensitivity of the fabricated sensor. In order to solve this problem, the differential centrifugal method was used to separate Fe NWs from other granular and rod-like impurities. Two different rotation speeds were selected for centrifugation, including 4,000 rpm and 2000 rpm. As presented in Figure 2C, the samples obtained by centrifugation at 4,000 rpm were composed of bulk Fe NWs, and some granular and rod-like impurities. The samples obtained by centrifugation at 2000 rpm shown in Figure 2D were all arranged and separated Fe NWs. The average diameter of Fe NWs is about 200 nm, and the average length of Fe NWs is about 200 μm. Therefore, only Fe NWs purified by centrifugation at 2000 rpm would be used for further investigation in wearable sensors.

### 3.2 Preparation and sensing mechanism analysis of flexible pressure sensors

**FIGURE 3:**
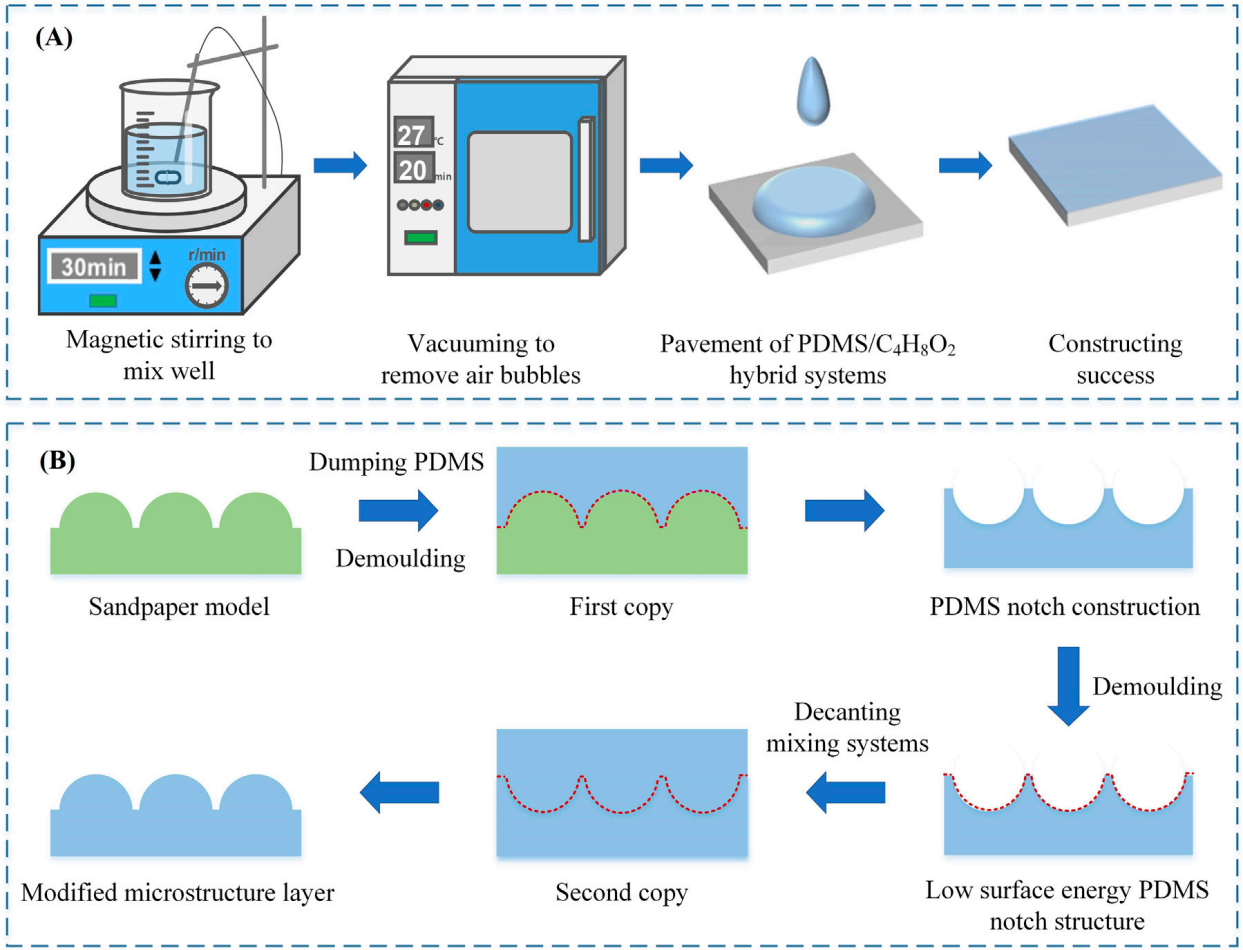
**(A)** Schematic diagram of the construction of the protective layer; **(B)** Schematic diagram of the construction of the modified microstructure layer.

PDMS was chosen as the base material for the protective layer of the flexible pressure sensor and the modified microstructure layer. To increase the fluidity of the protective layer in the preparation process without destroying its flexibility, a certain proportion of C_4_H_8_O_2_ was added to obtain a PDMS/C_4_H_8_O_2_ (V_PDMS_: V_C4H8O2_ = 7:3) hybrid system. The specific construction process of the protective layer is shown in Figure 3A. The main purpose of adding modified microstructures to the flexible pressure sensor is to enhance its own mechanical and sensing properties. The main steps in the construction of the modified microstructure layer are shown in Figure 3B.

**FIGURE 4:**
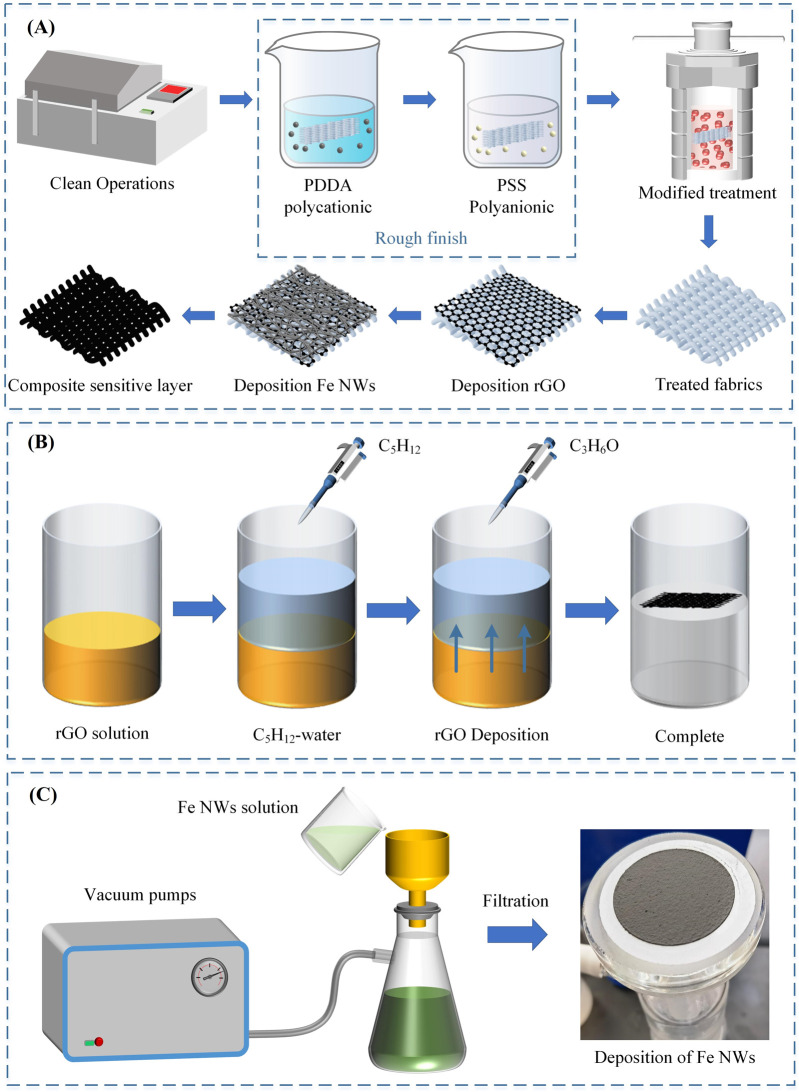
**(A)** Construction of the rGO/Fe NWs composites sensitive layer; **(B)** Deposition of rGO by self-assembly; **(C)** Deposition of Fe NWs by vacuum filtration technique.

Cotton and polyester fabrics were chosen as carriers for the composite sensitive layer of the flexible pressure sensor, and rGO/Fe NWs composites were deposited on both fabrics to construct the composite sensitive layer. To enhance the adhesion between the rGO/Fe NWs composites and the fabric carriers, the fabric carriers were polished with sandpaper to enhance the surface roughness. As shown in Figure 4A, the rGO/Fe NWs composites were then deposited onto the fabric carrier using ultrasonic treatment, self-assembly method, and vacuum filtration techniques, respectively. In Figure 4B, the rGO dispersion aqueous solution was centrifuged at 4,500 rpm/min for 40 min, and then 9,000 rpm for 40 min to remove the impurities from the aqueous solution. A simple, low-cost, and effective vacuum extraction deposition technique was proposed to prepare Fe NWs, which achieved the solid-liquid separation of Fe NWs by using negative vacuum pressure as the driving force, thus successfully depositing Fe NWs on the fabric carrier, as shown in Figure 4C.

**FIGURE 5:**
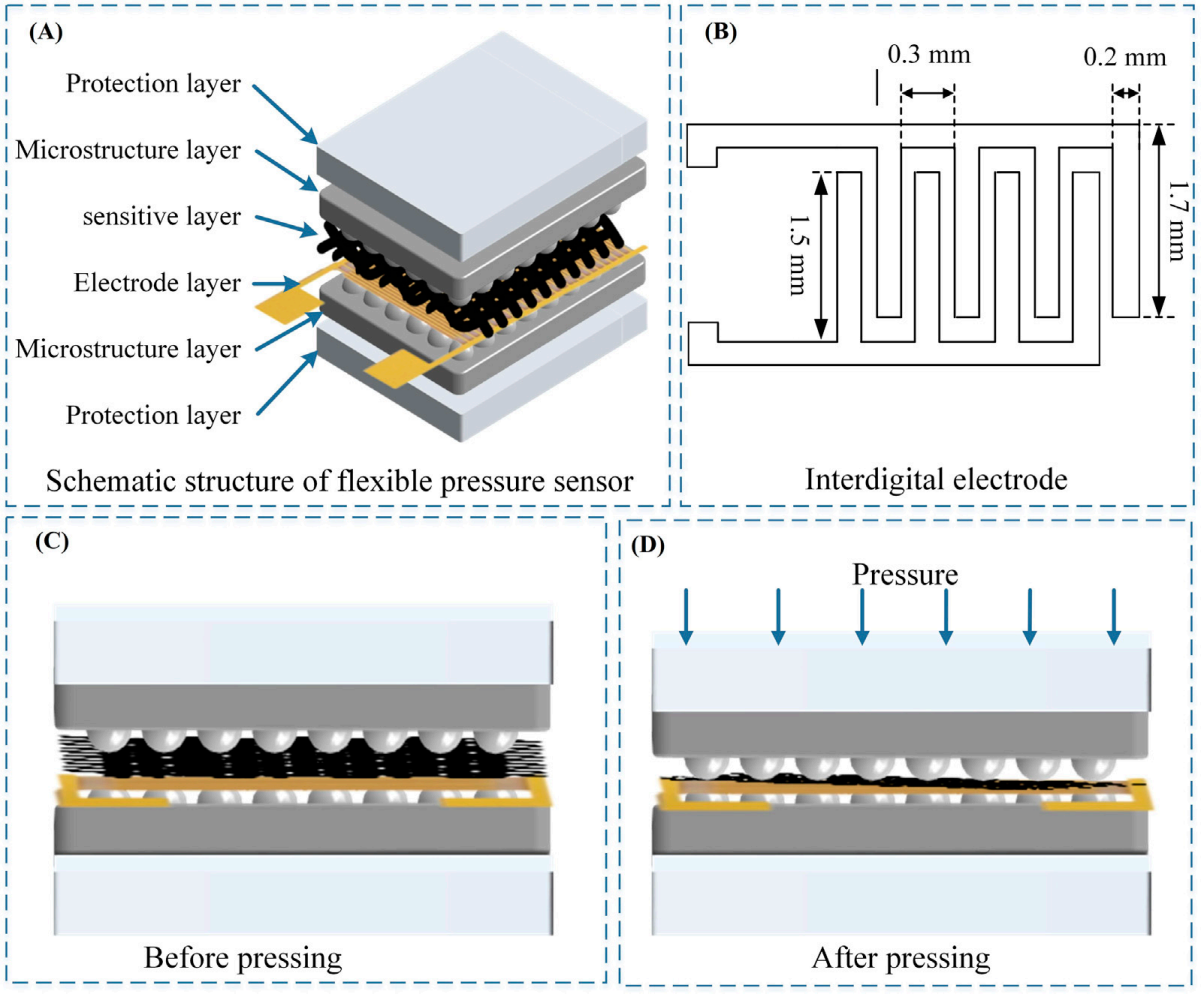
**(A)** Structural design of the flexible pressure sensor; **(B)** Interdigital electrode configuration; **(C)**–**(D)** The status of sensors before and after external pressure.

The overall schematic structure of the rGO/Fe NWs composites based flexible pressure sensor is shown in Figure 5A, which benefits from obtaining a flexible pressure sensor with high sensitivity, small size, and good flexibility. The designed flexible pressure sensor is of sandwich structure with length, width, and thickness of 2 cm, 2 cm, and 0.2 mm, respectively. The transmission electrode configuration has a significant impact on the sensing performance of the resistive flexible pressure sensor. Based on the position relationship between the electrode and the composite sensitive layer, the conductive silver pasted copper foil was used to form an interdigital electrode as the transmission electrode configuration of the flexible pressure sensor. The design of the interdigital electrode was composed of two interlocking brush-like electrodes, and one lead wire for connecting the external signal acquisition device. The size of a single brush-like electrode is shown in Figure 5B.

The cross-section of the flexible pressure sensor is shown in Figures 5C, D. An extruded hemispherical modified microstructure was formed from the surface of the upper and lower duplicated sandpaper. When the external pressure increased gradually, the composite sensitive layer would deform due to the compression of the hemispherical modified microstructure, and the resistance of the composite sensitive layer would decrease. When the external pressure gradually decreased, the hemispherical structure of the modified microstructure rose, and the rGO/Fe NWs composites sensitive layer gradually returned to the initial state, and its resistance gradually increased to the original size. In this way, flexible pressure sensors could sense external pressure. This sensing mechanism vividly showed that the compact flexible pressure sensor could obtain good flexibility, mechanical properties, and sensing properties based on the composite sensitive layer and the modified microstructure layer.

**FIGURE 6:**
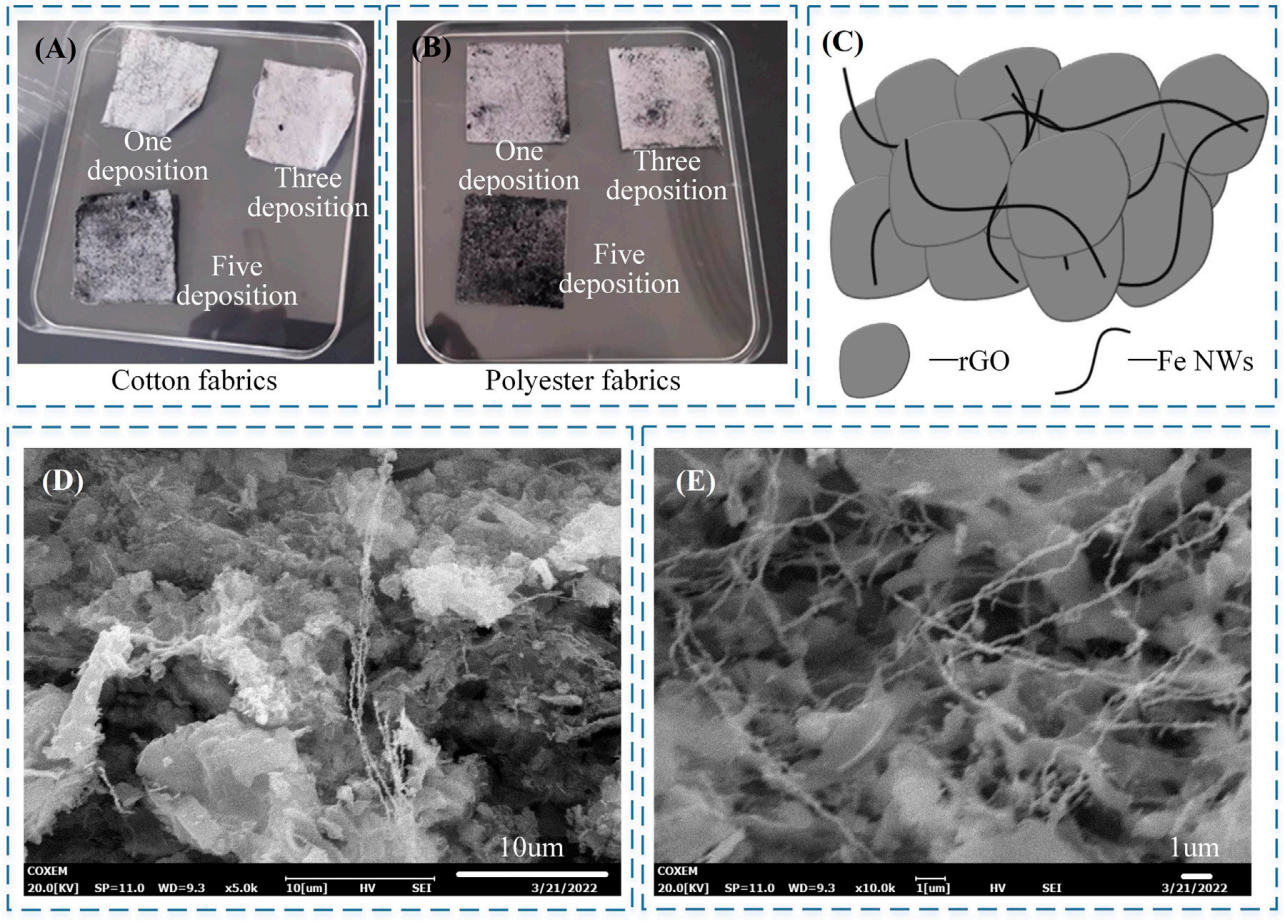
**(A)**–**(B)** rGO/Fe NWs composites sensitive layer; **(C)** composition diagram of the rGO/Fe NWs composites; **(D)**–**(E)** SEM of the sensitive layer with 5 times deposition of rGO/Fe NWs on cotton and polyester fabric.

Figures 6A–D show the rGO/Fe NWs composites based flexible pressure sensor by deposition one, three, or five times of rGO/Fe NWs solution on cotton or polyester fabrics. However, only the sensor prepared by five depositions showed better press-sensitive properties. Figure 6C shows the internal composition diagram of the rGO/Fe NWs composites. To maintain the excellent mechanical and sensing properties of the flexible pressure sensor, the sensitive layer of the composite pressure-sensitive material deposited five times would be used to assemble the flexible pressure sensor. The fabric carrier material has a great influence on the deposition effect. Figures 6D, E show that the polyester fabric had a fabric-like structure formed by Fe NWs and rGO in the SEM image after deposition, while it was rarely seen in the cotton fabric. From the microscopic point of view, this phenomenon showed that rGO/Fe NWs composites were deposited on polyester fabric very successfully, and the resulting composite sensitive layer was deposited flat and with good mechanical properties, therefore only polyester fabric would be used for the subsequent construction of the composite sensitive layer.

### 3.3 Pressure sensing performance of rGO/Fe NWs based flexible pressure sensor

**FIGURE 7:**
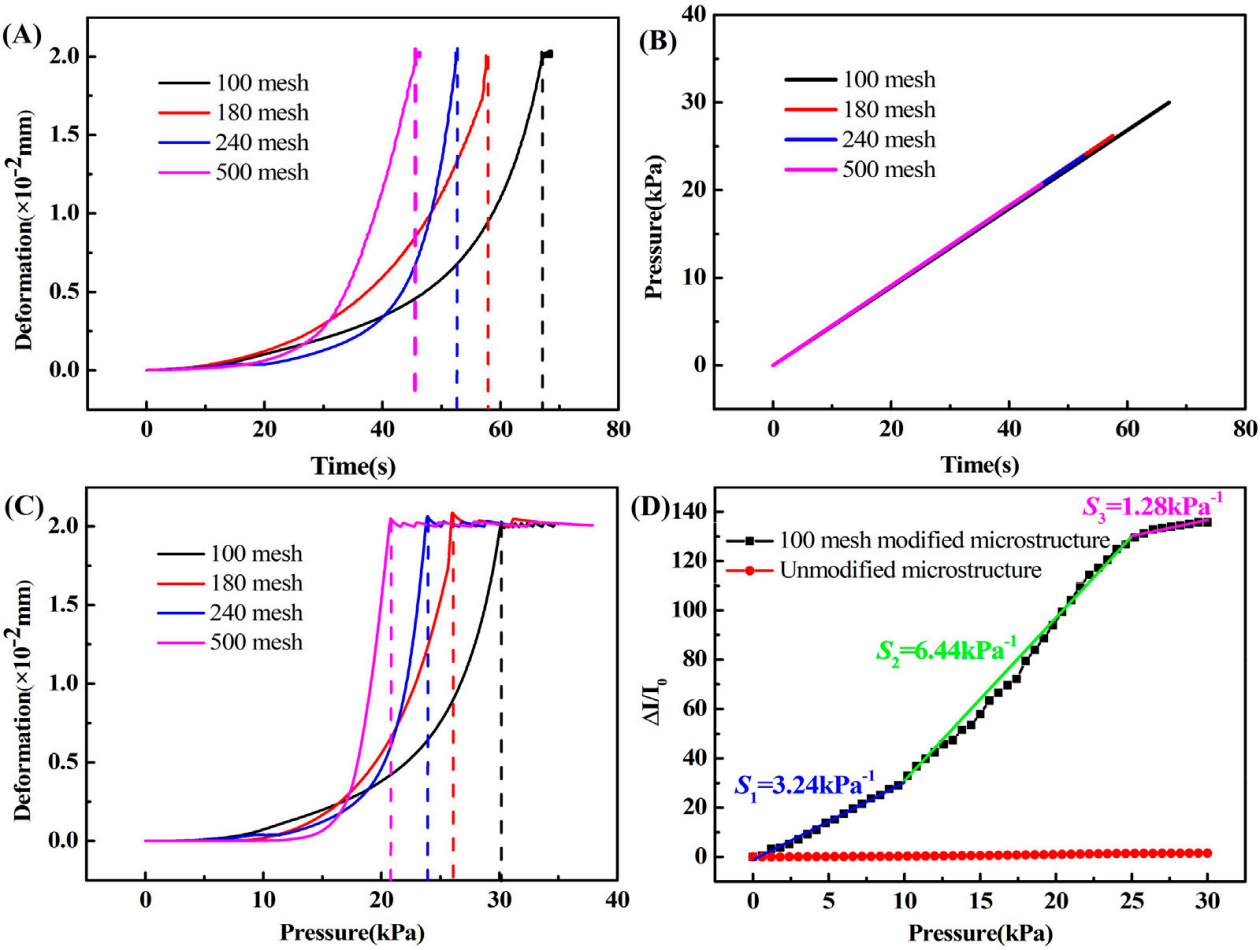
**(A)**–**(C)** The mechanical properties of the flexible sensor, and **(D)** the sensitivity of the flexible sensor.

The mechanical properties are an important indicator for flexible pressure sensors. Figure 7A shows the overall deformation of the flexible pressure sensor with time when it is subjected to a linearly increasing pressure. Figure 7B shows the curve of the applied pressure as a function of time. It could be noticed that the external pressure applied to the four flexible pressure sensors was increasing at a constant rate and in the same amount. Figure 7C is obtained by processing the data in Figures 7A, B, which shows the curves of deformation with pressure, where the four curves represent flexible pressure sensors with 100, 180, 240, and 500 mesh microstructure sizes respectively. When a linear pressure was applied at a constant rate with time, Figure 7C shows the same deformation curve as Figure 7A. The mechanical properties of the 100-mesh microstructure flexible pressure sensor in this investigation were the best, with the deformation reaching the limit state only when the pressure was 30 kPa. Therefore, only the 100 mesh rGO/Fe NWs composites based flexible pressure sensor would be used subsequently for relevant sensing performance tests and applications.

The sensitivity of the flexible pressure sensor can be expressed by the following equation [33].

$$S=\frac{I-I_{0}}{I_{0}∆P}=\frac{∆I}{I_{0}∆P}$$

where *S* represents the sensitivity of the flexible pressure sensor, *I*_0_ represents the current value of the flexible pressure sensor in its initial state, *I* represents the real-time current value through the flexible pressure sensor after applying pressure, Δ*P* represents the externally applied pressure value directly above the flexible pressure sensor, and Δ*I* represents the real-time current difference through the flexible pressure sensor. Figure 7D shows the current variation vs pressure curves for two types of rGO/Fe NWs composites based flexible pressure sensors with and without the 100-mesh modified microstructure. It could be observed that the sensitivity of the flexible pressure sensor with the addition of the 100-mesh roughness microstructure was much greater than that of the flexible pressure sensor without the microstructure. The current variation of the flexible pressure sensor with the 100-mesh modified microstructure increased as the externally applied pressure increased, and the entire curve could be roughly divided into three stages, including stage I (0–10 kPa) with a s sensitivity of *S*_1_ = 3.24 kPa^-1^, stage II (10–25 kPa) with a sensitivity of *S*_2_ = 6.44 kPa^-1^, and stage III (25–30 kPa) with a sensitivity of *S*_3_ = 1.28 kPa^-1^.

In stage I, only a small pressure was required to deform the sensitive material, due to the modified microstructure above and below the composite sensitive layer. This deformation would result in a large area of the composite sensitive material coming into contact with the electrode layer, forming a rapid change in contact resistance. In stage II, the pressure increased further and the modified microstructure layers above and below were squeezed to further intensify the contact between the composite sensitive material and the electrode surface. In stage III, with the continuous increase of pressure, the increase rate of the contact area between the composite sensitive layer and the transmission electrode layer decreased gradually, resulting in a decrease in sensitivity compared to the previous value.

**FIGURE 8:**
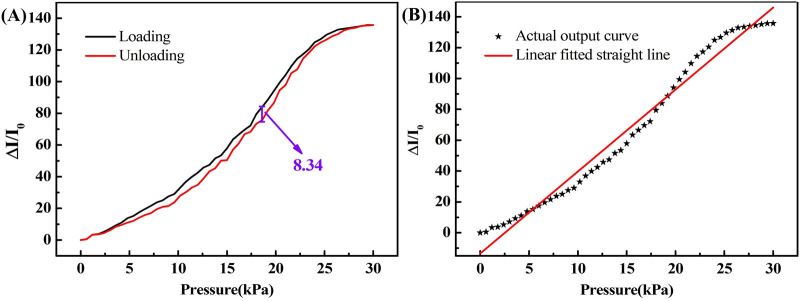
**(A)** Hysteresis, and **(B)** Linearity of flexible pressure sensor.

The hysteresis performance of the rGO/Fe NWs composites based flexible pressure sensor is shown in Figure 8A, where 8.34 was the maximum difference between the output of the flexible pressure sensor with a 100-mesh modified microstructure during loading and unloading. The hysteresis phenomenon was probably due to the elasticity and buffering effect of the deformable protective layer, which also proved that there was a strong interaction between the rGO/Fe NWs composites and the protective layer. Moreover, the strong interaction and the stable conductive pathway established by rGO/Fe NWs composites made the flexible pressure sensor show superior sensing performance. The following equation defines the hysteresis ratio (Huang et al., 2023),

$$\gamma_{H}=\frac{{\Delta \varphi }_{\max}}{\varphi_{\max}-\varphi_{\min}}\times 100\%$$

where *γ*_H_ is the hysteresis characteristic parameter of the flexible pressure sensor, Δ*φ*_max_ is the maximum difference between the output when loaded and unloaded, *φ*_max_ is the maximum value of the output, and *φ*_min_ is the minimum value of the output.

Then the hysteresis characteristic parameter described by Figure 8A could be calculated, which was 6.14%, indicating that the hysteresis characteristic of the flexible pressure sensor was very small and it would not affect the sensitivity, accuracy, and reliability of the flexible pressure sensor.

In general, there is always an error between the fitted curve and the actual output curve (Shi et al., 2018). In this investigation, the maximum residual between the actual output curve and the linear fitted line over the full range was 13.45.

**FIGURE 9:**
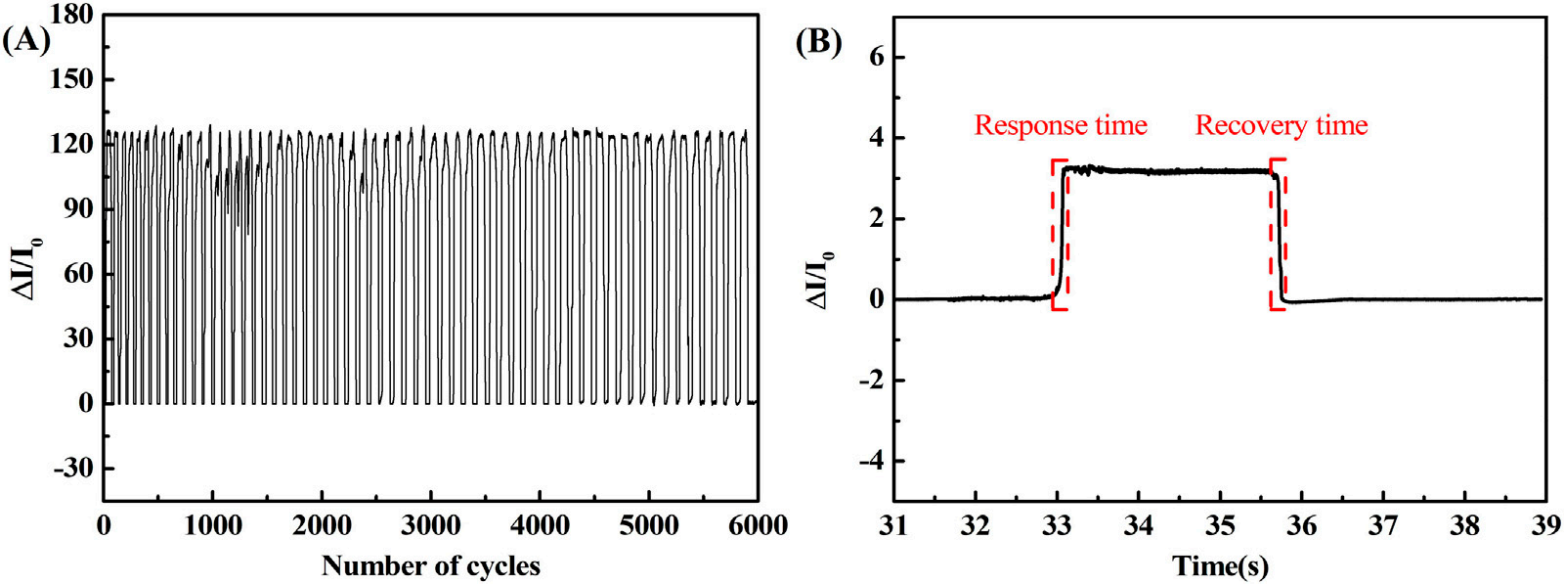
**(A)** Repeatability, and **(B)** response characteristics of flexible pressure sensor.

As shown in Figure 9A, repeatability evaluation was carried out on the rGO/Fe NWs composites based flexible pressure sensor with a 100-mesh modified microstructure. By applying a constant pressure of 30 kPa with 6000 load-unload repetitions, the current variation through the flexible pressure sensor was essentially uniform after a continuous period of operation, verifying excellent repeatability. As shown in Figure 9B, when 1 kPa pressure was applied and removed from the flexible pressure sensor, the response and recovery time were only 18 min and 20 min, respectively. Therefore, the flexible pressure sensor exhibited good stability and quick repose capabilities.

**FIGURE 10:**
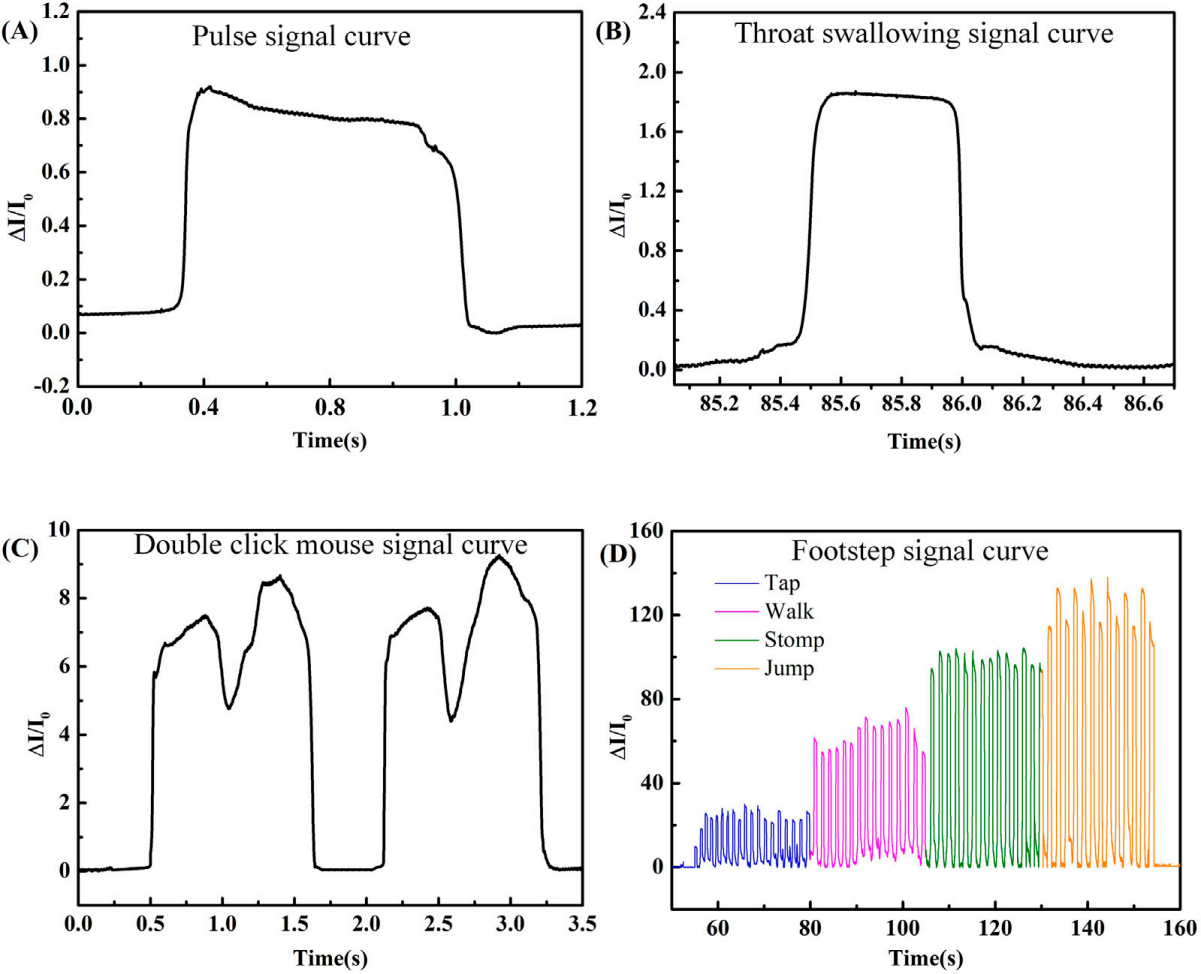
Response of flexible pressure sensor in **(A)** wrist pulse, **(B)** throat swallow, **(C)** mouse double-clicking, and **(D)** walking.

As illustrated by Figure 10, the flexible pressure sensor was further applied to detect human activities including the human wrist pulse, throat swallowing, mouse-clicking, as well as walking. The human wrist pulse signal could be detected by fitting the flexible pressure sensor tightly near the wrist artery, which acted as an important physiological signal reflecting the health status of the body. As shown in Figure 10A, a 75 min/stroke wrist pulse signal with a certain regularity was measured based on the pulse signal. As shown in Figure 10B, the flexible pressure sensor could be tightly attached to the human throat for swallowing tests with excellent flexibility and sensitivity. Figure 10C shows the response of the flexible pressure sensor upon mouse double-clicking. The sensor achieved two sharp peaks with fast response speed. When the first click ended, the sensor continued to perform the newly received signal instead of returning to the starting point. After the end of double-clicking, the sensor could completely return to its original signal status. Figure 10D shows the application of the flexible pressure sensor in walking status detection. The detection signal showed the intensity of status, namely, tap < walk < stomp < jump. Consequently, the flexible pressure sensor had high sensitivity and compatibility in human motion detection, laying a good foundation for human health applications.

## 4 Conclusion

In this study, a flexible pressure sensor based on rGO/Fe NWs composites was developed, featuring a compact size, high flexibility, good mechanical properties, and excellent sensing performance. This was achieved through the optimization of pressure-sensitive materials and the design of sensor’s microstructure. The flexible pressure sensor, featuring a 100-mesh modified microstructure, demonstrated an outstanding real-time response to both high and low pressures with excellent repeatability. The sensitivity of the sensor was segmented into three stages: stage I (0–10 kPa) *S*_1_ = 3.24 kPa^-1^, stage II (10–25 kPa) *S*_2_ = 6.44 kPa^-1^, and stage III (25–30 kPa) *S*_3_ = 1.28 kPa^-1^. The hysteresis characteristic was 6.14%, and the response/recovery time was 18 min and 20 min. For low-pressure applications, the flexible pressure sensor could be used to detect the wrist pulse signal, throat swallowing, and mouse double-clicking for remote human-computer interaction system applications. For high-pressure applications, human body walking activity can be detected, enabling self-health management. In summary, the flexible pressure sensor shows great potential for applications in the wearable field, health management, human activity, and human-computer interaction.

## Data Availability

The original contributions presented in the study are included in the article/supplementary material, further inquiries can be directed to the corresponding author.
